# Intermediate compartment (IC): from pre-Golgi vacuoles to a semi-autonomous membrane system

**DOI:** 10.1007/s00418-018-1717-2

**Published:** 2018-09-01

**Authors:** Jaakko Saraste, Michaël Marie

**Affiliations:** Department of Biomedicine and Molecular Imaging Center (MIC), University of Bergen, Jonas Lies vei 91, 5009 Bergen, Norway

**Keywords:** Secretory pathway, Golgi ribbon, ER–Golgi intermediate compartment (IC), Endosome, COPI, Rab1/Ypt1

## Abstract

Despite its discovery more than three decades ago and well-established role in protein sorting and trafficking in the early secretory pathway, the intermediate compartment (IC) has remained enigmatic. The prevailing view is that the IC evolved as a specialized organelle to mediate long-distance endoplasmic reticulum (ER)–Golgi communication in metazoan cells, but is lacking in other eukaryotes, such as plants and fungi. However, this distinction is difficult to reconcile with the high conservation of the core machineries that regulate early secretory trafficking from yeast to man. Also, it has remained unclear whether the pleiomorphic IC components—vacuoles, tubules and vesicles—represent transient transport carriers or building blocks of a permanent pre-Golgi organelle. Interestingly, recent studies have revealed that the IC maintains its compositional, structural and spatial properties throughout the cell cycle, supporting a model that combines the dynamic and stable aspects of the organelle. Moreover, the IC has been assigned novel functions, such as cell signaling, Golgi-independent trafficking and autophagy. The emerging permanent nature of the IC and its connections with the centrosome and the endocytic recycling system encourage reconsideration of its relationship with the Golgi ribbon, role in Golgi biogenesis and ubiquitous presence in eukaryotic cells.

## Introduction

The intermediate compartment (IC)—a dynamic membrane system consisting of peripheral and central domains—functions in protein sorting and bi-directional trafficking at the ER–Golgi interface of mammalian cells. The IC elements associate with ER exit sites (ERES), where they receive newly synthesized proteins and lipids from COPII-coated vesicles budding off the ER membrane. While it is generally accepted that the subsequent anterograde delivery of ER-derived cargo from ERES to the *cis*-face of the Golgi apparatus depends on motor-dependent movements of the IC elements along cytoskeletal filaments, the mechanisms of cargo transfer at the end stations of the ER–Golgi itinerary—at ERES and *cis*-Golgi—remain poorly understood (Hanna et al. 2018; Lorente-Rodriquez and; Barlowe 2011; Saraste and Marie 2016; Zanetti et al. 2012). This has left key questions regarding the nature of the IC and its role in the secretory pathway unanswered. As a result, although the existence of the IC as a discrete pre-Golgi entity is well recognized, it is still debated whether it represents a transient or permanent structure.

However, the currently prevailing model of the IC emphasizes its transient character. Accordingly, the IC is viewed as a collection of pleiomorphic transport intermediates that arise at ERES via the coalescence of ER-derived COPII vesicles, acquire Golgi-like properties as they move to the cell center and, finally, upon their arrival at the *cis*-face of the Golgi stacks assume a flat cisternal shape. Supporting this view, the IC elements recruit COPI coats, which are known to function in the sorting and retrograde transfer of selected components, such as lipids, cargo receptors and escaped ER proteins, to the ER (Rabouille and Klumperman 2005). Extensive membrane recycling by COPI vesicles is thought to alter the composition of the IC elements, resulting in their gradual transformation into *cis*-Golgi *cisternae*. Indeed, an analogous COPI-dependent maturation process is the favoured mechanism for cargo progression across the Golgi stacks to the *trans*-Golgi network (TGN) (Nakano and Luini 2010). However, the exclusive retrograde function of COPI vesicles at the level of the IC and the Golgi stacks has been challenged by results suggesting anterograde role(s) for the COPI coats (Hosobuchi et al. 1992; Love and Kreis, 1998; Orci et al. 1997; Park et al. 2015; Pepperkok et al. 1993; Peter et al. 1993; Malsam et al. 2005). To complicate matters further, COP-independent tubular intermediates have been implicated in retrograde transport at the ER–Golgi boundary (Bottanelli et al. 2017; Heffernan and Simpson 2014; Sengupta et al. 2015).

The organization of the ER–Golgi interface varies in different eukaryotic cells (Brandizzi and Barlowe 2013). In vertebrate cells, the ER network extends throughout the entire cytoplasm, while the Golgi stacks are positioned near the cell center, linked together into a continuous ribbon around the centrosome (Gosavi and Gleeson 2017). Thus, a large proportion of the ERES—in cultured mammalian cells about half of them—are found at the cell periphery, whereas the rest associate with the central IC elements residing next to the Golgi ribbon (Bannykh et al. 1996; Hammond and Glick 2000; Stephens 2003). A precondition of this spatial arrangement is the long distance movement of the IC elements from peripheral ERES to the Golgi region along microtubule (MT) tracks. A different organization of the early secretory pathway is encountered in invertebrates (e.g., *D.melanogaster* and *C. elegans*), plants and fission yeasts (e.g., *P. pastoris*). In these cells, the individual Golgi stacks remain separate and reside next to the widespread ERES, establishing compact secretory units for short-range ER–Golgi communication (Brandizzi and Barlowe 2013). Strikingly, the secretory system of the budding yeast *S. cerevisiae* consists of tubular networks, but lacks Golgi stacks (Rambourg et al. 2001; Suda et al. 2018). These findings have led to the idea that the IC developed late in evolution to solve the logistics problems posed by the large size of vertebrate cells (Brandizzi and Barlowe 2013). Its apparent absence in many eukaryotes, such as the important model organism *S. cerevisiae*, has contributed to the view of the IC as a transient structure rather than a discrete organelle.

However, the proposed compartmental diversity of the early secretory pathway is not in agreement with the conservation of protein machineries that operate in ER–Golgi trafficking across the eukaryotic kingdom (Barlowe and Miller 2013; Bonifacino and Glick 2004; Klute et al. 2011; Lee et al. 2004). Indeed, recent electron microscopic (EM) tomography analysis has suggested the presence of the IC in the compact secretory system of *C. elegans* (Hanna et al. 2018; Witte et al. 2011), and certain cell types in *D. melanogaster* lack Golgi stacks, but contain IC-like tubulovesicular membrane clusters (Kondylis and Rabouille 2009). Moreover, it has been suggested that plants contain a *cis*-Golgi compartment, which is analogous to the IC and functions in the biogenesis of the Golgi stacks (Day et al. 2013; Donohoe et al. 2013; Ito et al. 2018). In general, defining compartments based on their transport machineries and the traffic patterns they support (Day et al. 2013), raises the possibility that the tubular *cis*-Golgi network of *S. cerevisiae* and the mammalian IC are equivalent structures as well (Kurokawa et al. 2014; Marie et al. 2008; Suda et al. 2018).

This review makes an attempt to provide the reader a concise summary on the structural, functional and dynamic properties of the IC in mammalian cells. We discuss recent data on novel functions of the IC that are not directly related to ER–Golgi trafficking. Since the nature of the IC remains a matter of dispute, it is of interest to take another look at the different models of this compartment, which are based on the employment of various reporters to visualize its dynamics in living cells. Interestingly, imaging of the IC during different stages of the cell cycle suggests that it—despite being dynamic—represents a stable organelle. Regarding the present discussion, of particular importance are the recently established permanent connections of the IC elements with the recycling endosomes and the centrosome (Bowen et al. 2017; Marie et al. 2009, 2012). Based on these findings, we propose a model on the role of the stable IC elements and recycling endosomes as “linker compartments” in the Golgi ribbon, operating in the biogenesis of the Golgi stacks. This model can also help to clarify the relationship of the secretory systems of mammalian and yeast cells.

However, since a historical sketch can be informative—particularly considering a cellular component as enigmatic as the IC—we start by recalling the developments that lead to the placement of this compartment on the map of the cell.

## The early days of the IC

The early 1980s marked an exciting period in the membrane traffic field. Endosomes had just been identified and initially characterized as intermediates in the pathway that leads from the plasma membrane (PM) to lysosomes (Mellman 2006). The striking geometry of endosomes—that is, their division into vacuolar and tubular domains—and their luminal acidification were implicated in the sorting of internalized molecules for recycling back to the PM or delivery for lysosomal degradation. Similarly, acidification turned out to be important in the secretory pathway, ensuring the correct sorting of secretory proteins in the *trans*-Golgi region to constitutive and regulated post-Golgi carriers (Burgess and Kelly 1987). Regarding pre-Golgi events, two-way traffic between the transitional ER elements—now commonly referred to as ERES—and the *cis*-Golgi was thought to involve small transport vesicles (Palade 1975). George Palade’s vesicle shuttle hypothesis became the paradigm for membrane traffic in general, gaining support, for example, from the demonstration of the role of clathrin-coated vesicles in endocytic uptake of molecules at the PM (Robinson 2015). Furthermore, ER–Golgi transport was considered an MT-independent short distance transport step, involving the transfer of newly synthesized proteins from the centrally located ERES to the nearby *cis*-Golgi *cisternae* (Kelly 1990).

**Fig. 1:**
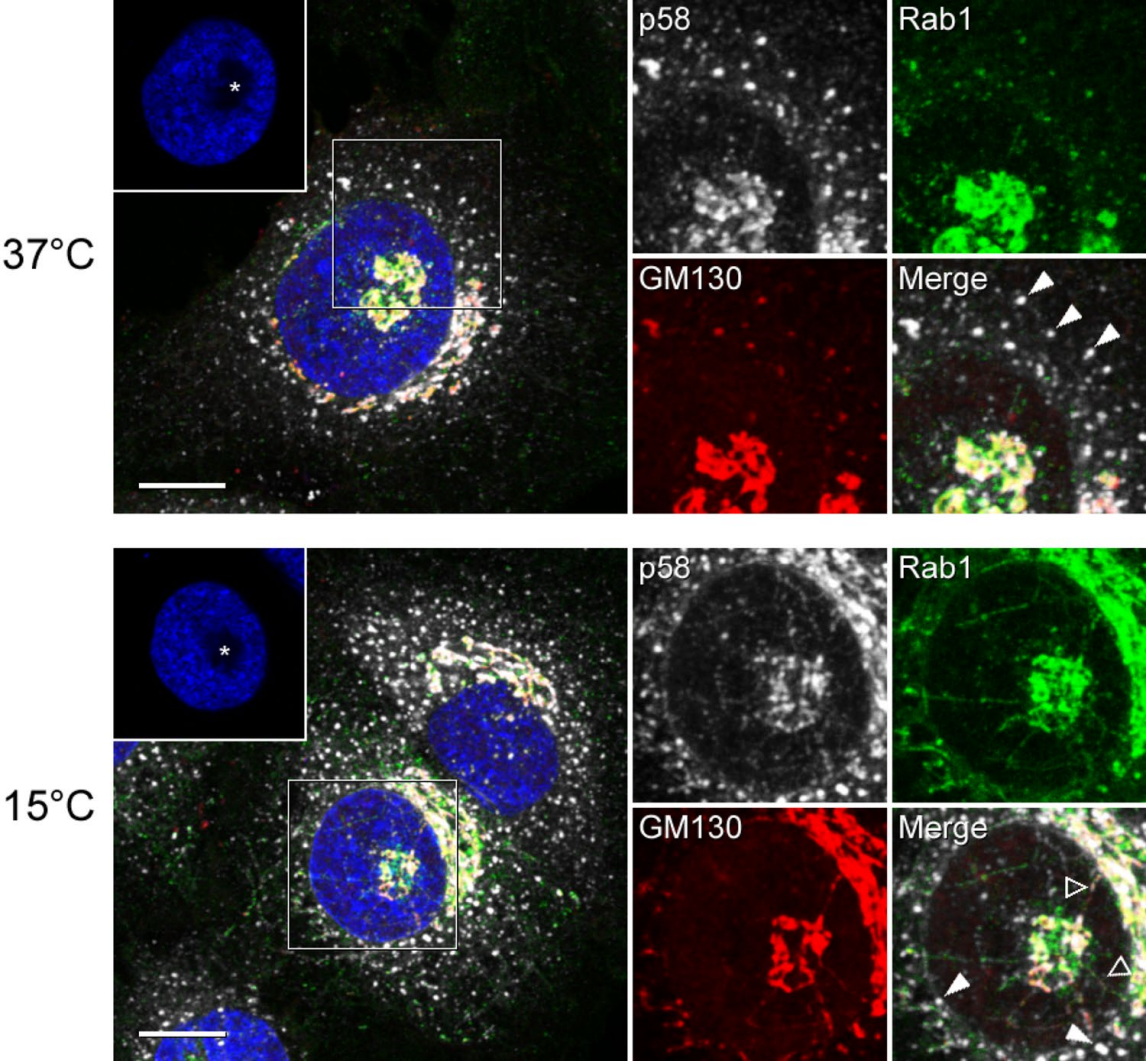
Distribution of the IC as studied by confocal microscopy. Normal rat kidney (NRK) cells stably expressing GFP-Rab1 were kept at 37 °C or shifted for 3 h to 15 °C, followed by staining with antibodies against p58 and GM130, as well as DAPI (to visualize the nuclei). The three IC/*cis*-Golgi proteins co-localize in the Golgi area, the pericentrosomal domain of the IC (BRC), as well as peripheral IC elements (white arrowheads) that pile up—or become more readily detectable—in the cells shifted to 15 °C. The overlap is not complete due to enrichment of these proteins to different IC subdomains. Low temperature also boosts the tubulation of the IC, for example, the formation of tubular connections between the BRC and the Golgi ribbon (open arrowheads). The insets with single DAPI staining highlight the nuclear “pockets” (asterisks), where the BRC resides after its relocation under the nucleus. The anti-GM130 antibodies were kindly provided by Francis Barr, Department of Biochemistry, University of Oxford, UK. Bars: 10 µm

An increased appreciation of the more complex membrane organization of the ER–Golgi boundary was the outcome of multiple lines of research (for reviews see Balch 1990; Bonatti and Torrisi 1993; Hauri and Schweizer 1992; Huttner and Tooze 1989; Lippincott-Schwartz 1993; Pelham 1989; Saraste and Kuismanen 1992). A key methodological development was the introduction of membrane viruses as tools in membrane traffic, allowing the fusion of genetics and morphology. Temperature-sensitive (ts) mutants of vesicular stomatitis (VSV ts-O45) and Semliki Forest virus (SFV ts-1), carrying reversible folding defects in their membrane glycoproteins, allowed the application of immunocytochemistry to follow the synchronized movement of the proteins from the ER to the PM, following the shift of the cells from restrictive to permissive temperature (Bergman et al. 1981; Saraste and Hedman 1983). As low-temperature incubation had been shown to affect specific steps of both endocytic and secretory transport (Marsh and Helenius 1980; Matlin and Simons 1983), it was of interest to combine such incubations with temperature shift experiments using viral ts-mutants to achieve more detailed mapping of the early secretory pathway. Interestingly, immuno-EM of SFV ts-1 infected cells shifted from 39 to 15 °C showed that the viral membrane proteins exit the ER, but accumulate in large vacuoles (up to 0.5 µm in diameter), tubules and vesicles both in the *cis*-Golgi region and more peripheral locations. Light microscopic (LM) studies revealed a scattered distribution of these structures throughout the cytoplasm, a pattern distinct from that of the ER and Golgi (Kuismanen and Saraste 1989; Saraste and Kuismanen 1984; see also Fig. 1). The SFV proteins arrested at 15 °C contained high-mannose oligosaccharides, indicating lack of processing by Golgi enzymes. Importantly, the transport block was reversible and the viral proteins entered the Golgi stacks within minutes after shifting the cells to permissive temperature, indicating that the vacuoles represent normal intermediates in the secretory pathway.

Biochemical and immunocytochemical studies showed that the transport of VSV tsO45 glycoproteins is similarly affected at 15 °C (Balch et al. 1986; Bonatti et al. 1989). In addition, temperature shift experiments with both VSV and SFV mutants provided evidence for the role of the tubular extensions of the pre-Golgi vacuoles in the entry of viral glycoproteins to the Golgi stacks (Saraste and Kuismanen 1984; Trucco et al. 2004). Indeed, as the pre-Golgi elements turned out to consist of interconnected vacuolar and tubular domains, resembling endosomes in morphology, the term “exosome” was proposed for these novel structures (Balch 1990; Saraste and Kuismanen 1984). In hindsight, taking into account the striking similarities of the IC and endosomal networks revealed by subsequent studies, it seems unfortunate that this term was not permanently adopted to designate these pre-Golgi structures, but instead refers today to the luminal vesicles of endosomes released from different cell types (van Niel et al. 2018).

Subsequent studies have shown that the transport of membrane and soluble secretory proteins is similarly affected at 15–16 °C (Blum et al. 2000; Saraste et al. 1986; Trucco et al. 2004). In general, ever since its introduction, numerous studies have employed the reversible 15–16 °C temperature block to follow trafficking or modification of proteins in the early secretory pathway, or to synchronize the passage of cargo across the Golgi stacks (Lavieu et al. 2013, 2014; Volchuk et al. 2000; Trucco et al. 2004). However, even today the precise mechanism of the low-temperature block remains unknown. A simple explanation is that temperature reduction, by slowing down transport in general, causes a traffic jam at crossroad sites, where multiple pathways meet (Kuismanen and Saraste 1989). However, the increased tubulation of membranes during 15–16 °C incubation or shortly after shifting cells to 37 °C (Ben-Tekaya et al. 2005; Blum et al. 2000; Martinez-Alonso et al. 2005; Palokangas et al. 1998; Simpson et al. 2006) suggests more specific effects on the transport machinery. Indeed, a recent study showed that the activation of Golgi-localized GTPases, such as Arf1, is inhibited at low temperature, resulting in the release of some of their effectors from membranes (Gilbert et al. 2018).

Independent proof for the existence of a distinct pre-Golgi compartment came from EM studies of coronavirus-infected cells showing the early budding of progeny virus particles into a smooth membrane compartment at the ER–Golgi interface (Tooze et al. 1984). Interestingly, initiation of O-glycosylation of the viral membrane (M) protein, by addition of N-acetyl-galactosamine (GalNAc), was suggested to take place in this novel compartment, making it reactive for the GalNAc-specific lectin *Helix pomatia* (Tooze et al. 1988; Krijnse-Locker et al. 1994). Subsequent studies verified that the coronavirus “budding compartment” corresponds to the IC (Klumperman et al. 1994) and also revealed that the intracellular site of virus maturation is determined by the retention of viral M protein at the *cis*-side of the Golgi stacks (Machamer et al. 1990). In addition, we know today that the enzyme responsible for the addition of GalNAc (GalNAcT2) cycles between the Golgi and the IC (Jarvela and Linstedt 2012), and under certain conditions—including perhaps viral infection—can accumulate in the latter compartment, making it strongly positive for *Helix pomatia* (Gill et al. 2010). Besides coronaviruses, the IC has been suggested to support the intracellular maturation of a number of virus types, including bunya-, entero-, flavi-, picorna- and vacciniaviruses (Hsu et al. 2010; Jäntti et al. 1997; Miller and Krijnse-Locker 2008; Risco et al. 2002).

**Fig. 2:**
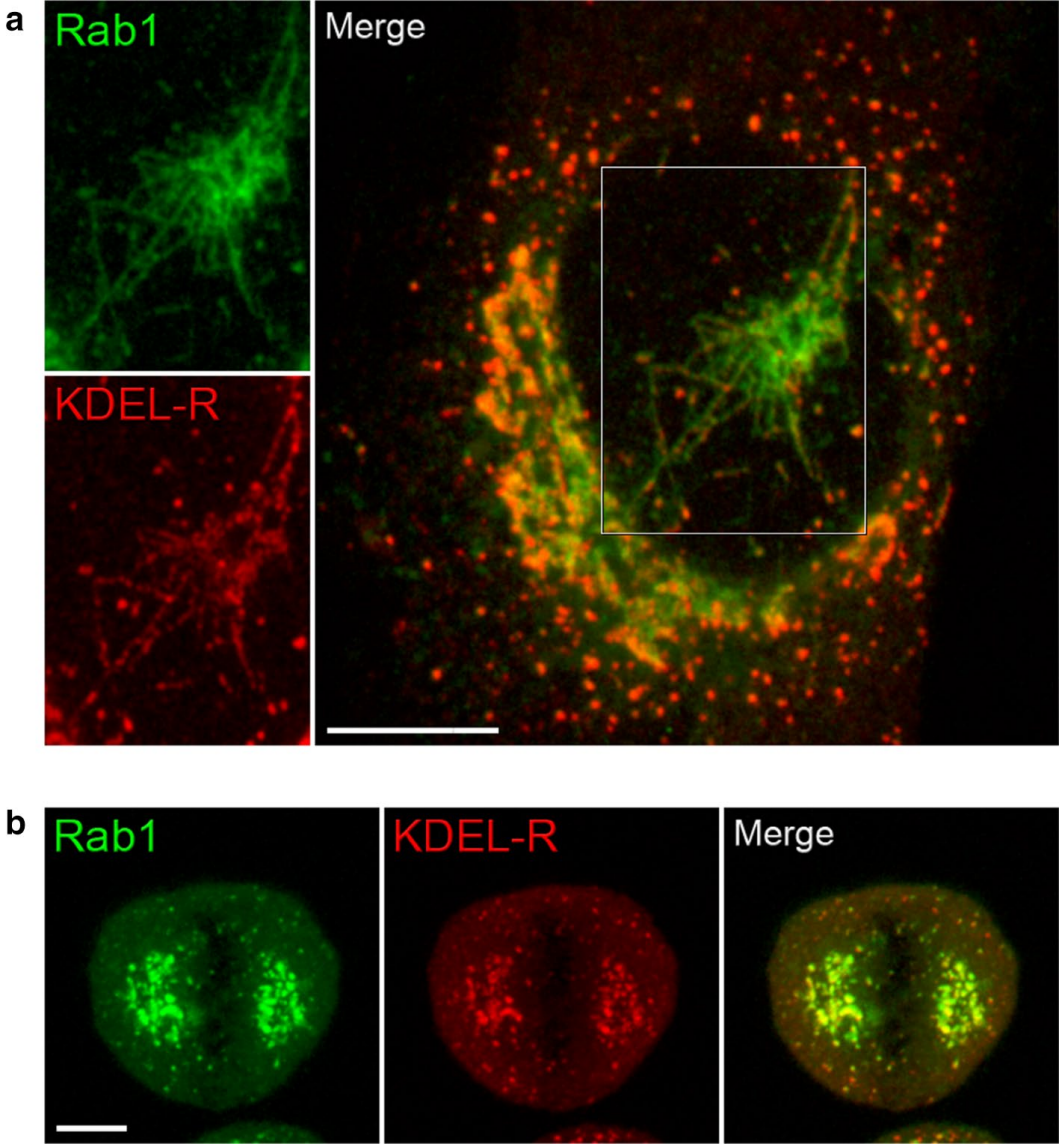
The KDEL-receptor localizes to the IC throughout the cell cycle. Confocal microscopic localization of the KDEL-receptor in GFP-Rab1-expressing NRK cells at interphase (**a**) or metaphase (**b**). During interphase, the receptor and Rab1 largely overlap in IC elements at the cell periphery and in the juxtanuclear Golgi region. Both proteins are also present in the BRC (framed area), but show preferential enrichment to its globular (vacuolar) and tubular subdomains, respectively. Notably, they also co-localize to mitotic IC elements, which maintain their spatial organization at metaphase, being present both at the cell periphery and in the pericentrosomal region close to the spindle poles. Antibodies against the KDEL-receptor were kindly provided by Irina Majoul, Institute of Biology, University of Lübeck, Germany. The images in (**b**) were reproduced from Marie et al. (2012). Bars: 10 µm

The discovery of the mechanism of retention of luminal ER proteins equipped with the C-terminal tetrapeptide KDEL-signal (HDEL in yeast) opened up for the operation of a special sorting compartment at the ER–Golgi boundary. Namely, it was suggested that these abundant molecular chaperones, such as BiP and PDI, are retained in a dynamic process that involves their continuous escape from the ER lumen and retrieval from a post-ER location (Munro and Pelham 1987; Pelham 1989). The 15^o^C/coronavirus budding compartment was proposed as the “salvage compartment” (Warren 1987) from which the escaped KDEL-proteins are returned to the ER. Furthermore, the attachment of the KDEL-signal to a lysosomal enzyme and the application of the 15 °C block gave evidence that the phosphotransferase initiating the formation of the mannose-6-phosphate lysosomal targeting signal resides in the IC (Pelham 1988; Lazzarino and Gabel 1988). Subsequently, the yeast and mammalian KDEL-receptors were identified (Lewis and Pelham 1990; Semenza et al. 1990) and the latter was found to predominantly localize to the IC/*cis*-Golgi (Griffiths et al. 1994; Tang et al. 1993; see also Fig. 2a). The recycling of the KDEL receptor at the ER–Golgi boundary is now known to take place in COPI vesicles (Majoul et al. 2001; Martínez-Menárguez et al. 1999; Orci et al. 1997). Similarly, integral membrane proteins that cycle between the ER and the IC/*cis*-Golgi were shown to contain dilysine (KKXX)-signals in their cytoplasmic C-terminal tails, which bind directly to COPI coats (Cosson and Latourneur 1997; Jackson et al. 1990; Nilsson et al. 1989).

In conclusion, by the end of the 1980s both morphological and functional data were available supporting the existence of a distinct sorting compartment at the ER–Golgi boundary. Notably, the post-ER retrieval of KDEL-proteins meant that to support specific ligand–receptor interactions such a pre-Golgi compartment had to maintain its own luminal conditions and therefore be discontinuous with the ER (Pelham 1989). Indeed, subsequent studies showed that the binding of the KDEL-ligands to their receptor prefers a slightly acidic pH (Wilson et al. 1993), indicating that pre-Golgi protein sorting is dependent on luminal acidification, in analogy to the sorting events taking place in endosomes and *trans*-Golgi. Subsequently, a number of studies have highlighted the importance of luminal acidification in two-way trafficking and quality control within the IC (Appenzeller-Herzog and Hauri 2004; Bu et al. 1995; Satoh et al. 1996). The luminal pH of the IC/*cis*-Golgi has been estimated to be between 6.0 and 6.7 (Vavassori et al. 2013). In addition, there is some evidence for a pH gradient, since only IC elements in the central Golgi area label with DAMP, a marker for acidic compartments (Palokangas et al. 1998).

## Identification of p58/ERGIC-53/LMAN1

An important turning point in the study of the IC was the identification of its first endogenous marker protein p58/ERGIC-53. Rat p58 was discovered with the help of a polyclonal antibody prepared against a *cis*-Golgi membrane fraction from pancreatic acinar cells, corresponding to the site of the 16 °C transport block (Saraste et al. 1986, 1987). The human homolog ERGIC-53 (89% identity) was identified in a screen of monoclonal antibodies generated against a Golgi fraction isolated from cultured epithelial cells (Schweitzer et al. 1988). The cytoplasmic tails of these non-glycosylated type I transmembrane proteins (Lahtinen et al. 1992, 1996; Schindler et al. 1993) are equipped with KKFF-signals, which by binding to COPII- and COPI-coats result in their cycling between the ER and IC/*cis*-Golgi (Kappeler et al. 1997; Tisdale et al. 1997). The recycling of p58/ERGIC-53 is inhibited when cells are kept at 15 °C (Fig. 1), resulting in the pile-up of the protein in the same pre-Golgi structures where the VSV and SFV glycoproteins are arrested (Plutner et al. 1992; Saraste and Svensson 1991; Schweitzer et al. 1990), verifying that the 15 °C block site is equivalent to the compartment defined by p58/ERGIC-53.

Sequence comparisons also revealed the identity of ERGIC-53 with a human mannose-binding protein MR60 (Arar et al. 1995). Accordingly, the luminal N-terminus of ERGIC-53 was shown to contain an L-type lectin domain that specifically binds high-mannose oligosaccharides in a calcium-dependent manner (Itin et al. 1996; Velloso et al. 2003; Zheng et al. 2013), hence the additional designation LMAN1 (lectin mannose-binding protein 1). Today, p58/ERGIC-53/LMAN1 is the best-characterized receptor for export of soluble glycoproteins from the ER (Appenzeller-Herzog et al. 1999). It employs both oligomerization (formation of dimers and hexamers) and accessory proteins (e.g., MCFD2 or ERp44) during its interaction with specific cargo, such as serum coagulation factors (Nichols et al. 1998; Zheng et al. 2013) or monomeric IgMs (Anelli and Sitia 2008; Cortini and Sitia 2010). Release of cargo from the receptor takes place in the low pH and calcium conditions prevailing in the lumen of IC/*cis-*Golgi (Appenzeller-Herzog et al. 2004).

**Fig. 3:**
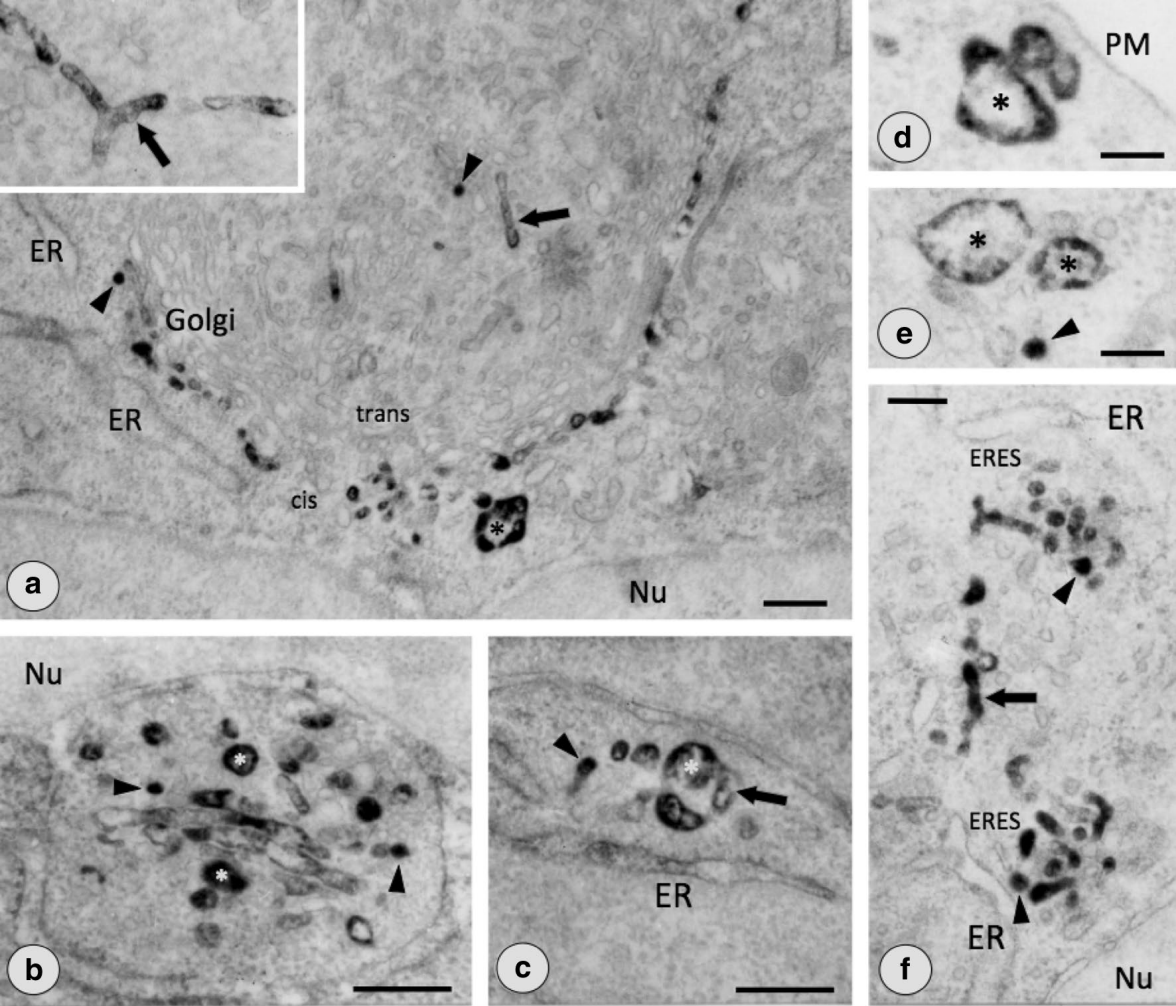
Ultrastructure of the IC as examined by immunoperoxidase EM. The pleiomorphic nature of the p58-containing IC elements is illustrated by images taken of NRK (**a**) and mouse myeloma cells (**b–f**), showing the presence of this recycling receptor in the *cis*-most Golgi cisterna, as well as small vesicles (arrowheads), narrow tubules (arrows) and large vacuolar structures (asterisks; diameter up to 0.5 µm). In addition to the *cis*-Golgi region, the latter are found throughout the cytoplasm, even close to the plasma membrane (PM). **f** p58-positive vesicular tubular clusters (VTCs) close to ER exit sites (ERES). ER, endoplasmic reticulum; Nu, nucleus. Bars: 0.5 mm (**a**–**c**), 0.25 mm (**d**–**f**). Reproduced from Saraste and Marie (2016) by permission from Elsevier. **f** Reproduced from Saraste et al. (1987)

Localization of p58/ERGIC-53/LMAN1 by immuno-EM gave new data on the ultrastructure of the IC. In addition to the *cis*-most Golgi *cisterna* and small vesicles and tubules, p58 was found in large pleiomorphic structures with endosome-like appearance (Horstman et al. 2002; Lahtinen et al. 1992; Saraste et al. 1987; Saraste and Svensson 1991; Ying et al. 2000; Fig. 3). The expansion of the latter most likely gives rise to the pre-Golgi vacuoles observed at 15 °C (Saraste and Kuismanen 1984; Trucco et al. 2004). However, other groups defined the IC as vesicular–tubular clusters (VTCs) that typically consist of small vesicles and tubules (see e.g., Fig. 3f), and are devoid of larger structures (Balch et al. 1994; Klumperman et al. 1998; Martínez-Menárguez et al. 1999). As discussed below, live imaging of IC dynamics indicates that the VTCs are stationary structures, while the vacuoles function as mobile cargo carriers within the IC system (Mironov et al. 2003). This could explain why many of the VTCs lack the large pleiomorphic IC component. Moreover, the existence of the latter is not only based on EM observations, but has been demonstrated by analytical differential centrifugation as well (Ying et al. 2000).

LM localization of p58 in different cell types confirmed the widespread distribution of the IC elements (Fig. 1; Saraste and Svensson 1991; see also; Lotti et al. 1992). Moreover, the striking disassembly of the Golgi stacks in cells treated with the newly introduced compound brefeldin A (BFA) was found to involve the formation long tubules, which mediate retrograde transport of Golgi enzymes—evidently via the IC—to the ER (Lippincott-Schwartz et al. 1990). Together, these studies provided the first indication that two-way communication at the ER–Golgi boundary occurs over long distances and depends on the integrity of the cytoskeletal microtubule (MT) tracks. The observed spatial organization of the IC also meant that ER-to-Golgi transport and endocytosis share the same cellular topology. Accordingly, the two types of transport carriers, endosomes and “exosomes”, were suggested to travel “side by side” from the periphery to the cell center and even communicate along the way (Saraste and Kuismanen 1992).

## Building IC domains: Rab1 and Arf1/COPI in action

Starting with the pioneering studies in the early 1980s, applying yeast genetics and biochemical dissection of mammalian cell-free systems (Bonifacino and Glick 2004; Lee et al. 2004), a wealth of information has accumulated on the molecular machineries that regulate transport in the early secretory pathway—including cargo receptors, COP coats, Arf and Rab GTPases, tethering factors and fusion proteins. Recent comprehensive reviews have addressed the functions of these molecular players in more detail (Barlowe and Miller 2013; Gillingham and Munro 2016; Gomez-Navarro and Miller 2016; Hutagalung and Novick 2011; Kaczmarek et al. 2018; Malsam and Söllner 2011; Szul and Sztul 2011; Zanetti et al. 2012). Here, we focus on two components of this machinery, Rab1 and COPI coats, which appear to preferentially associate with different subdomains of the IC and play important roles in bi-directional ER–Golgi trafficking.

### Rab1-positive tubules

GTPases of the Rab family—Ypt proteins in the yeast *S. cerevisiae*—are master regulators of membrane traffic. In their active membrane-bound conformation, they interact with multiple effectors, thereby affecting the assembly, motility, tethering or fusion of transport carriers. Moreover, by recruiting peripheral proteins and lipid-modifying enzymes to membranes, Rab proteins are thought to organize the formation of membrane domains, thereby having a major impact on organelle structure and identity (Behnia and Munro 2005; Stenmark 2009). The functions of the different compartment-specific Rab proteins acting in the secretory and endocytic pathways are coordinated by guanine nucleotide exchange factors (GEFs) and GTPase-activating proteins (GAPs) that regulate the switch between their active (GTP-bound) and inactive (GDP-bound) states, thereby ensuring the directionality of transport. The three Rab proteins operating in the yeast secretory pathway, Ypt1 (yeast counterpart of Rab1), Ypt31/32 (Rab11) and sec4 (Rab8), provide an example of such a Rab cascade (Lipatova et al. 2015; Mizuno-Yamasaki et al. 2012). Recent studies support the idea that both yeast and mammalian cells contain two conserved transport protein particle (TRAPP) complexes—TRAPPIII and TRAPPII—which can act as GEFs during the activation of Ypt1 (Rab1) and Ypt31/32 (Rab11) (Riedel et al. 2017; Thomas et al. 2018). However, although the successive functions of the TRAPPs in the yeast secretory pathway is relatively well established (Suda et al. 2018), the roles of their mammalian counterparts remain less clear. One complication is that yeast Ypt31/32 is considered a component of the *trans*-Golgi/TGN, whereas its mammalian ortholog Rab11 associates with the endocytic recycling compartment (ERC)—also referred to as recycling endosomes.

Rab1/Ypt1 is the best-known Rab protein acting in the early secretory pathway (Goud et al. 2018; Lipatova et al. 2015; Saraste 2016). Mammalian Rab1 is an abundant GTPase (Gilchrist et al. 2006) that specifically associates with the cytoplasmic side of pre-Golgi membranes, being present throughout the IC network (Griffiths et al. 1994; Marie et al. 2012; Saraste et al. 1995; Satoh et al. 2003). Live imaging showed the striking oscillatory recruitment of GFP-Rab1 to IC elements, their subsequent movement from ERES either to the Golgi region or the cell periphery and the bi-directionality of both pathways (Sannerud et al. 2006). It also revealed the association of Rab1 with an extensive network of narrow tubules, which are in dynamic continuity with the “globular” (vacuolar) parts of the IC (Fig. 2, see below, Fig. 5). Notably, this tubular system expands during neuronal differentiation, suggesting its possible biosynthetic functions (Sannerud et al. 2006).

The preference of Rab1 for the tubular domain of the IC also resulted in the uncovering of the centrosomal connection of this compartment. Due to centrosome re-positioning in motile cells, or at the onset of mitosis (see below, Fig. 6), a pool of Rab1-positive membranes separates from the Golgi ribbon and forms a tubular meshwork at the cell center (Marie et al. 2009; Mochizuki et al. 2013; Figs. 1, 2). In migrating cells, this event may be required for the rapid delivery of components to the cell’s leading edge, while the mitotic event most likely initiates the partitioning of the IC (Marie et al. 2012; Dale et al. in preparation). Due to its similarity with the pericentrosomal ERC, we designated this central domain of the IC as the biosynthetic recycling compartment (BRC; Saraste and Goud 2007). The localization of the KDEL-receptor to these membranes (Fig. 2) is in accordance with their key role in membrane recycling.

Many of the effectors of Rab1—such as p115, GM130 and giantin—function in membrane tethering, suggesting its participation in multiple transport steps (Goud et al. 2018; Saraste 2016). Accordingly, like yeast Ypt1, Rab1 regulates bi-directional ER–Golgi trafficking (Galea et al. 2015; Kamena et al. 2008; Plutner et al. 1991), in agreement with the presence of both antero- and retrograde cargo in IC tubules (Palokangas et al. 1998; Simpson et al. 2005). In addition, Rab1 has been implicated in intra-Golgi traffic (Plutner et al. 1991), although this function may not be readily compatible with its reported localization to the IC/*cis*-Golgi. Rab1 also plays an important in role Golgi biogenesis (Haas et al. 2007; Goud et al. 2018). Knockdown of Rab1 leads to Golgi fragmentation (Aizawa and Fukuda 2015; Galea et al. 2015), while microinjection of Rab1 mutants causes complete Golgi disassembly (Wilson et al. 1994). As discussed below, Rab1 (or one of its two isoforms) may also regulate pathways that bypass the Golgi stacks.

### COPI-coated vacuoles

Like Rab1, COPI coats preferentially associate with the IC/*cis*-Golgi (Griffiths et al. 1995; Oprins et al. 1993; Orci et al. 1997). For example, in professional secretory cells about 70% of the coats associate with the pre-Golgi VTCs (Martínez-Menárguez et al. 1999). In addition, COPI is involved in vesicle formation at the lateral rims of the Golgi stacks (Duden et al. 1991; Rabouille and Klumperman 2005). The binding of COPI coats to membranes is regulated by GTPases of the Arf family. Interestingly, Arf1—the master COPI regulator at the IC/*cis*-Golgi—is also involved in the recruitment of clathrin adaptor proteins (APs) at the *trans*-Golgi/TGN (Kaczmarek et al. 2018). The GEFs activating Arf1 at the *cis*- and *trans*-sides of the Golgi—GBF1 and BIG1/BIG2, respectively—are the targets for BFA (Mansour et al. 1999; Togawa et al. 1999). Besides Arf1, also Arf4 and Arf5 have been implicated in ER–Golgi trafficking (Ben-Tekaya et al. 2010; Chun et al. 2008; Volpicelli-Daley et al. 2005), suggesting the operation of different types of COPI vesicles (Popoff et al. 2011) at the level of the IC. In line with this possibility, the pericentrosomal BRC that separates from the Golgi ribbon contains a distinct pool of COPI coats (Marie et al. 2009).

As mentioned earlier, the general idea is that the COPI vesicles mainly function in retrograde transport (Rabouille and Klumperman 2005). Interestingly, however, blocking GBF1 function—by knock-down or using the specific inhibitor Golgicide A—arrests the VSV G-protein in the IC, suggesting a role of COPI coats in anterograde IC-to-Golgi transport (Manolea et al. 2008; Sáenz et al. 2009). Also, recent studies demonstrated the involvement of COPI-coated tubules in anterograde transport of VSV G protein at the level of the Golgi stacks (Park et al. 2015; Yang et al. 2011). Furthermore, besides generating vesicles, COPI coats could contribute to the organization of the IC elements (Bonifacino and Lippincott-Schwartz 2003). In this respect it is of interest that they seem to preferentially associate with the vacuolar IC domains (Horstmann et al. 2002; Presley et al. 2002; Sannerud et al. 2006; see below).

In conclusion, Rab1 and Arf1/COPI could play important roles in the formation of vacuolar and tubular IC domains, which play distinct roles in trafficking. The dynamic continuity of these subdomains (see below) suggests that their formation and functions are highly co-ordinated. Accordingly, Rab1 has been reported to participate in COPI-dependent vesicular (or tubular) transport (Alvarez et al. 2003; Gilchrist et al. 2006; Monetta et al. 2007), and inhibition of COPI function typically results in the proliferation of the tubular IC domain (Ben-Tekaya et al. 2010; Marie et al. 2009; Szul et al. 2007). Obviously, this is a simplistic scenario and future studies are expected to reveal a number of additional factors, such as lipid-modifying enzymes (Bechler et al. 2011; Ben-Tekaya et al. 2010), which greatly impact IC organization. In addition, cytoskeletal filaments are required for the remodeling of the IC elements, as highlighted by recent studies of WHAMM, an activator of actin nucleation, which is recruited to IC tubules by Rab1 and also interacts with MTs. (Campellone et al. 2008; Russo et al. 2016).

Finally, the preferential pre-Golgi localization of Rab1 and COPI in mammalian cells emphasize the compositional and functional similarity of the IC with the Ypt1-containing *cis-*Golgi compartment in the yeast *S. cerevisiae*. Taking into account that Rab proteins define organelle identity, it is of interest that Rab1 can functionally replace Ypt1 in yeast (Haubruck et al. 1989). In addition, COPI coats turn out to play analogous roles in the two compartments by ensuring membrane recycling and organelle maturation (Papanikou et al. 2015).

## Modeling the IC

The static images generated by the early EM studies, employing transported cargo or the newly identified resident proteins as IC markers (see, e.g., Fig. 3), left ample room for interpretation and opened up for different ideas on the nature of this compartment. For example, the IC was considered a subdomain of the ER (Krijnse-Locker et al. 1994; Sitia and Meldolesi 1992) or part of a *cis*-Golgi network (CGN), a tubular compartment analogous to the TGN operating in membrane recycling at the entry face of the Golgi stacks (Mellman and Simons 1992). The CGN concept rapidly gained popularity, probably explaining why it took some time—close to 20 years—before the IC became more generally accepted as a distinct pre-Golgi structure, as evidenced by its entry into the textbooks (Alberts et al. 2002). This development was due to the introduction of new methodology, allowing, e.g., the coupling of the synchronizable VSV tsO45 G-protein with green fluorescent protein (GFP) to visualize pre-Golgi membrane dynamics in living cells (Presley et al. 1997; Scales et al. 1997). These pioneering studies revealed the translocation of large pre-Golgi structures (up to 1 µm in diameter) along MT tracks, mediating long-distance transfer of cargo between peripheral ER sites and the central Golgi apparatus. They also demonstrated in vivo the division of these structures into globular (vacuolar) and tubular subdomains (Presley et al. 1998, see below). Subsequently, a number of fluorescent reporters have been used to visualize ER-to-Golgi transport (Verissimo and Pepperkok 2013). However, despite all the efforts, the nature of the IC remains a matter of dispute. The various fluorescent markers employed may highlight different aspects of IC dynamics due to their differential enrichment in the two IC subdomains. In addition, some may not faithfully recapitulate the behavior of their endogenous counterparts. Nevertheless, live imaging has provided the basis for three different models of the IC, as discussed in the following.

### Model A: transient transport carriers

**Fig. 4:**
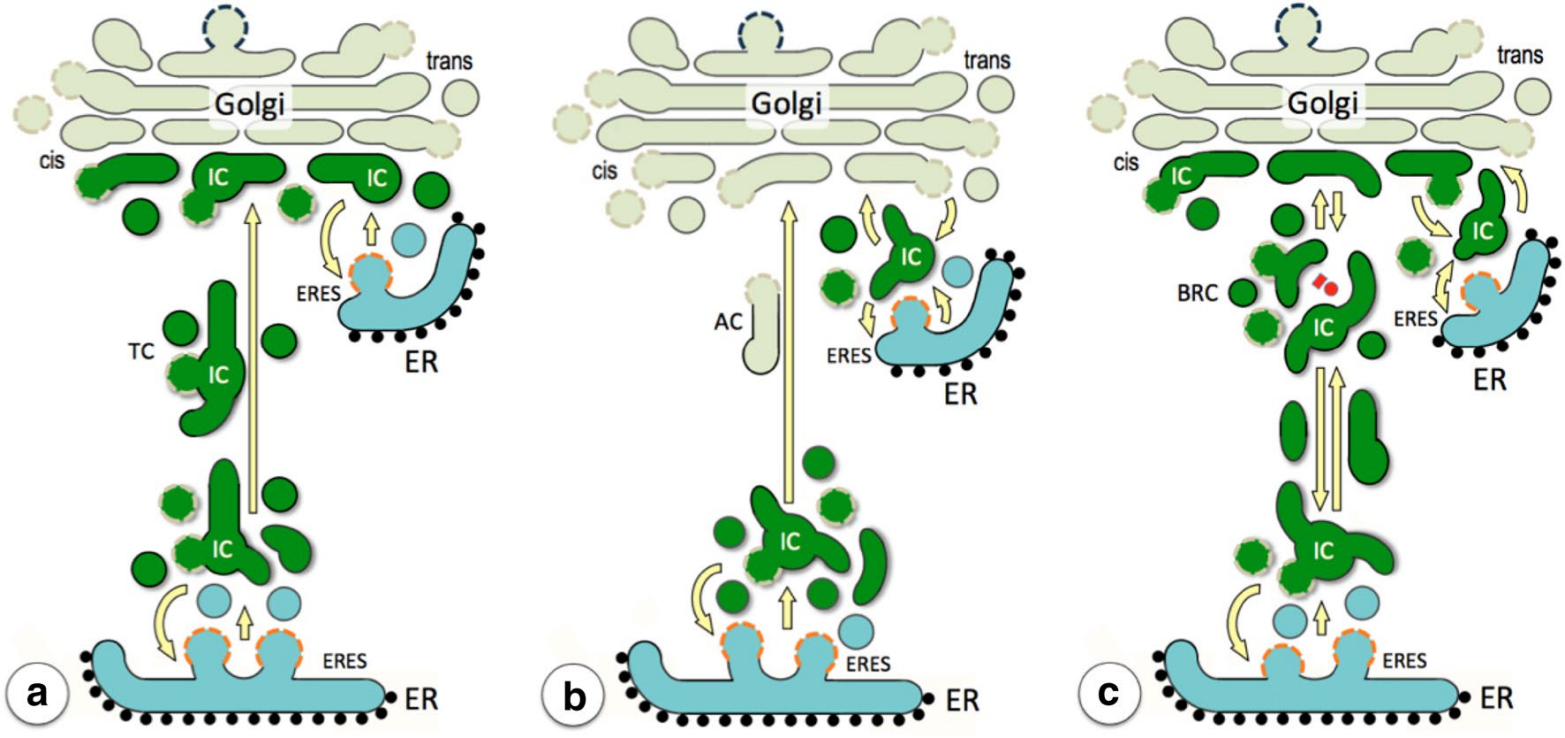
Different views of the IC in mammalian cells. All three models depict the existence of peripheral and central, Golgi-adjacent ERES. They focus on the role of the IC in ER–Golgi trafficking and do not illustrate “lateral” communication between the IC elements residing next to ERES. **a** The IC elements act as transport carriers. According to this model the IC consists of vesicular tubular clusters (VTCs) that form de novo at ERES via homotypic fusion of COPII vesicles (orange coats). The VTCs are mobilized in bulk and move in a MT-dependent stop-and-go fashion—most likely from ERES to ERES—to the *cis-*Golgi region. These mobile transport carriers (TCs) can change their composition by budding COPI vesicles (gray coats). Depending on whether the Golgi stacks are stationary or maturing structures, the TCs either fuse with or transform into *cis*-Golgi cisternae (as shown here). The latter event would involve homotypic fusion between the IC elements. **b** A stable IC residing next to ERES. Here the IC is thought to consist of ERES-associated, long-lived membrane clusters that are relatively immobile and maintain bi-directional communication with the ER and *cis*-Golgi. Accordingly, ER-to-Golgi transport requires two heterotypic fusion events. The first involves the fusion of COPII vesicles with pre-existing IC elements, and the second the delivery of newly synthesized cargo to the *cis-*Golgi by IC-derived anterograde carriers (ACs). **c** A permanent network of dynamic IC elements. This model combines key aspects of the previous models by considering that two types of IC components—vacuoles and tubules, which display distinct dynamics—mediate long-distance communication within a permanent IC network. Thus, relatively stationary vacuoles anchored at ERES are interconnected with other peripheral (not shown) or central vacuoles via dynamic tubules. Due to this MT-dependent communication, vesicle-mediated exchange of material with the ER and the recruitment of proteins from the cytosol, the vacuoles mature, become mobile, change shape and move along MT tracks between neighboring ERES (not shown), as well as between peripheral ERES and the Golgi region. The vacuoles arriving at the cell center are anchored at the non-compact regions that connect the Golgi stacks (see Fig. 7). Thus, these membranes are normally distributed at multiple sites across the Golgi ribbon, but, due to their MT-dependent connection with the centrosome (red), can also establish an extensive compartment (BRC), as the latter separates from the Golgi ribbon (as shown here). Communication within this IC network, including the events taking place at *cis*-Golgi, is expected to involve homotypic fusions between vacuolar, tubular and vesicular IC elements. Adapted from Saraste and Marie (2016). By permission from Elsevier

The currently prevailing model—and accordingly the one presented in many textbooks—views the pleiomorphic IC elements as transient cargo carriers (Fig. 4a). This model has its roots in the early studies on the dynamic pre-Golgi elements containing membrane-bound or soluble cargo, such as VSV G-protein or procollagen (Presley et al. 1997; Scales et al. 1997; Shima et al. 1999). In this model the IC elements are first assembled at ERES in process that involves the homotypic fusion of ER-derived COPII vesicles. They then move in a saltatory (“stop-and-go”) fashion to the Golgi region where they appear to merge with the Golgi stacks. In essence, the IC elements are transient structures that are continuously made de novo at ERES and consumed at *cis*-Golgi.

Since this model presents the pre-Golgi elements as cargo carriers themselves, they have also been referred to as transport complexes (TCs; Bannykh and Balch 1997; Stephens and Pepperkok 2001). Indeed, the well-documented size and shape heterogeneity of the vacuolar, tubular and vesicular IC elements (Fig. 3; Ying et al. 2000) opens up for their function as different types of carriers—“cars and trucks” (Fan et al. 2003)—with the ability to accommodate different types of large or small cargo, such as procollagen fibers, lipoprotein particles, soluble or membrane-bound proteins or lipids. Interestingly, carrier formation at ERES for large-sized cargo turns out to involve a highly complex interplay between the COPII machinery, IC membranes, and novel organizers, such as TGF and TANGO (Hanna et al. 2018).

It should be noted that the TC model is based on the assumption that the mobile pre-Golgi carriers that can be resolved by LM correspond to the large-sized VTCs. However, a number of studies have documented that these elements become elongated as they start to move (Marie et al. 2009; Presley et al. 1997, 2002; Sannerud et al. 2006). Since the VTCs are unlikely to be capable of such a shape change, it may be important to make a distinction between stationary VTCs residing at ERES and large vacuolar IC elements that are deformable for transport purposes (Lippincott-Schwartz et al. 1998; Fig. 3).

The long distance saltatory movements of the IC elements from ERES to the cell center—at a speed of about 1 µm/s—take place on the MTs radiating from the centrosome (Gurel et al. 2014). This centralization process depends on the minus-end directed MT motor dynein that associates with the IC membranes (Burkhardt et al. 1997; Presley et al. 1997; Roghi and Allan 1999; Watson et al. 2004). A good candidate for the receptor that links dynein to IC elements is the Arf1 effector golgin-160, a long coiled-coil tethering protein that localizes to the *cis*- and lateral sides of the Golgi stacks. Namely, besides recruiting dynein to central Golgi elements, golgin-160 is also required for anterograde ER-to-Golgi transport (Yadav et al. 2012). Moreover, its dissociation from membranes during mitosis coincides with inhibition of the centralization of IC elements that still interact with spindle MTs (Marie et al. 2012; Yadav et al. 2012). Notably, the IC also contains the plus-end directed motor kinesin (Lippincott-Schwartz et al. 1995; Tomás et al. 2010), which could be involved in retrograde transport, the shaping of the vacuolar and tubular elements or their positioning at ERES and/or Golgi region (Brown et al. 2014). A special class of MTs that are nucleated at *cis*-Golgi membranes (Rivero et al. 2009) has been implicated in the formation of the Golgi ribbon. Whether these non-centrosomal MTs have additional roles in ER–Golgi trafficking remains unknown.

### Model B: stationary ERES-associated membrane clusters

Live imaging of GFP-coupled ERGIC-53 in HeLa cells showed its preferential localization to long-lived stationary structures, providing a basis for an alternative model of the IC (Ben-Tekaya et al. 2005). According to this model, the IC is a stable compartment consisting of a constant number of tubulovesicular clusters (VTCs) that reside in the vicinity of the widely distributed ERES (Klumperman et al. 1998). These clusters communicate with the ER and *cis*-Golgi with the help of distinct transport carriers (Appenzeller-Herzog and Hauri 2006). Accordingly, ER-derived COPII vesicles first undergo heterotypic fusion with the stable IC elements, whereafter the latter generate a second class of anterograde carriers (ACs) that separate from the stationary IC clusters and move along MT tracks to the *cis*-Golgi (Fig. 4b). Surprisingly, while GFP-ERGIC-53 was detected in mobile tubules that “laterally” connect the IC clusters (Ben-Tekaya et al. 2010), it was absent from the Golgi-directed ACs, despite the fact that the endogenous p58/ERGIC-53 can reach the *cis*-Golgi *cisternae* (Schweitzer et al. 1988; Klumperman et al. 1998; Fig. 3). However, the ACs contained a soluble reporter (signal sequence-containing DsRed; Ben-Tekaya et al. 2005), suggesting that they represent authentic cargo carriers.

The stable IC model received support from studies on a neuronal GABA transporter, indicating that the protein contains a special motif required for its exit from the IC (Farhan et al. 2008). Furthermore, previous observations on the presence of COPI coats on motile pre-Golgi carriers (Presley et al. 2002; Scales et al. 1997; Shima et al. 1999) lead to the proposal that these coats organize the formation of the novel ACs (Appenzeller-Herzog and Hauri 2006). In this respect, the stable IC model is also in accordance with previous ideas on the dual function of COPI in antero- and retrograde trafficking at the level of the IC (Lowe and Kreis 1998). It is also supported by the early results concerning the effect of BFA on the localization of p58/ERGIC-53. Namely, upon COPI removal by BFA Golgi proteins redistribute to the ER, whereas p58/ERGIC-53 remains associated with apparently stable IC structures at ERES (Fullekrug et al. 1997; Lippincott-Schwartz et al. 1990; Saraste and Svensson 1991; Ward et al. 2001).

### Model C: permanent network of dynamic vacuoles and tubules

The most recent model of the IC elaborated here (Fig. 4c)—based on live imaging of GFP-Rab1 dynamics in NRK cells (Marie et al. 2009, 2012; Sannerud et al. 2006; Saraste and Marie 2016)—combines the dynamic and stationary aspects of the two previous models. It retains the idea that the IC elements themselves function as mobile transport carriers (Model A). However, instead of moving from ERES to *cis*-Golgi these vacuolar and tubular carriers, which display distinct dynamics, are suggested to operate in the context of an interconnected IC network. Thus, despite being highly dynamic, the IC is considered as a permanent organelle. An advantage of this model is that it can take into account both the extensive evidence of the dynamics of the IC elements and recent data that speaks in favor of their stability.

**Fig. 5:**
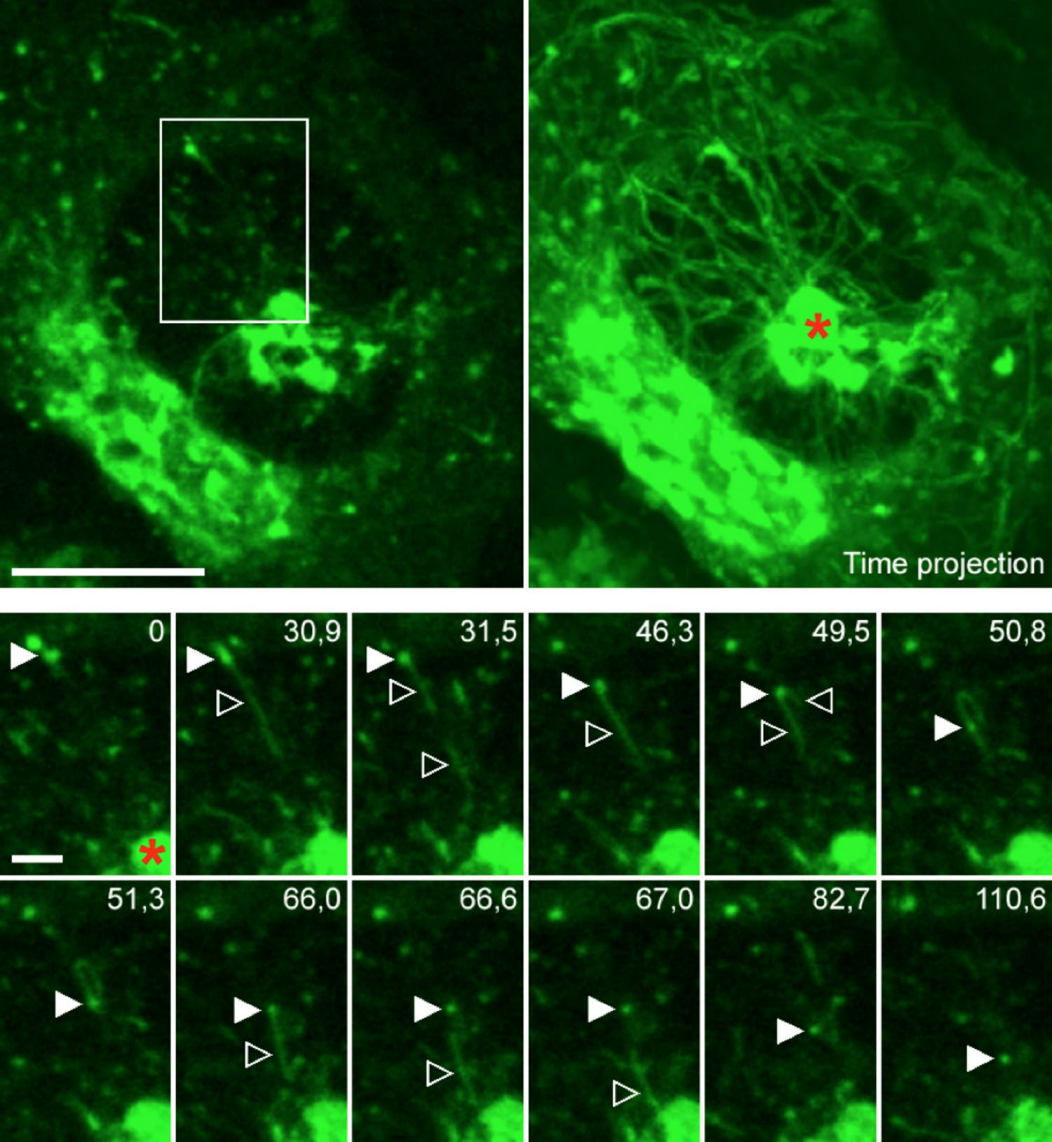
Dynamics of the IC network as studied by spinning disk confocal microscopy. The images are derived from a movie taken from a GFP-Rab1-expressing NRK cell displaying a BRC (asterisk), which has separated from the Golgi ribbon. The time projection highlights three types of tracks related to the MT-dependent movements of the IC elements between (i) peripheral ERES, (ii) peripheral ERES and the BRC, and (iii) the BRC and the Golgi ribbon. Selected images (time = seconds) corresponding to the framed area, showing the motility of both globular (vacuolar) and tubular IC elements. A globular structure (white arrowhead) continuously sheds narrow tubules (open arrowheads), while it itself also moves in a stop-and-go fashion toward the cell center BRC (asterisk). These events also appear to involve its movement as a varicosity (*bolus*) along the tubule it first extends. Bars: 10 µm (upper panels), 2 µm (bottom panels). Adapted from Marie et al. (2009)

As discussed above, Rab1 binds to the cytoplasmic side of the vacuolar and tubular IC elements, with some preference for the tubular domain. Previously, the two endogenous IC markers Rab1 and p58/ERGIC-53 were shown to display overlapping intracellular distributions (Sannerud et al. 2003; Saraste et al. 1995; Fig. 2). Accordingly, fluorescent GFP-Rab1 was found to associate with two types of elements in living cells, evidently corresponding to those highlighted also by GFP-ERGIC-53. Firstly, it bound to large ERES-associated structures that remain stationary for variable periods of time. However, most of them eventually become mobile, change shape—become elongated—and move toward the Golgi region or the cell periphery (Marie et al. 2009; Sannerud et al. 2006). As pointed out earlier, by being deformable they must correspond to vacuoles rather than VTCs. Second, GFP-Rab1 was localized to highly dynamic long tubules that protrude from the vacuoles, pinch off and—just like the vacuoles—display bi-directional mobility along MTs, moving either to the Golgi region, or in the opposite direction, toward the cell periphery (Fig. 5). Despite the direction, the vacuolar and tubular dynamics takes place in a stop-and-go fashion, i.e., from ERES to ERES. An interesting additional feature revealed by GFP-Rab1 imaging was *bolus* structures, that is, vacuolar structures that develop a long tubule and then appear to slide along it (Marie et al. 2009; Fig. 5). Similar mobile *boluses* have been previously described in the endosomal system (Ayala 1994; Hopkins et al. 1990).

**Fig. 6:**
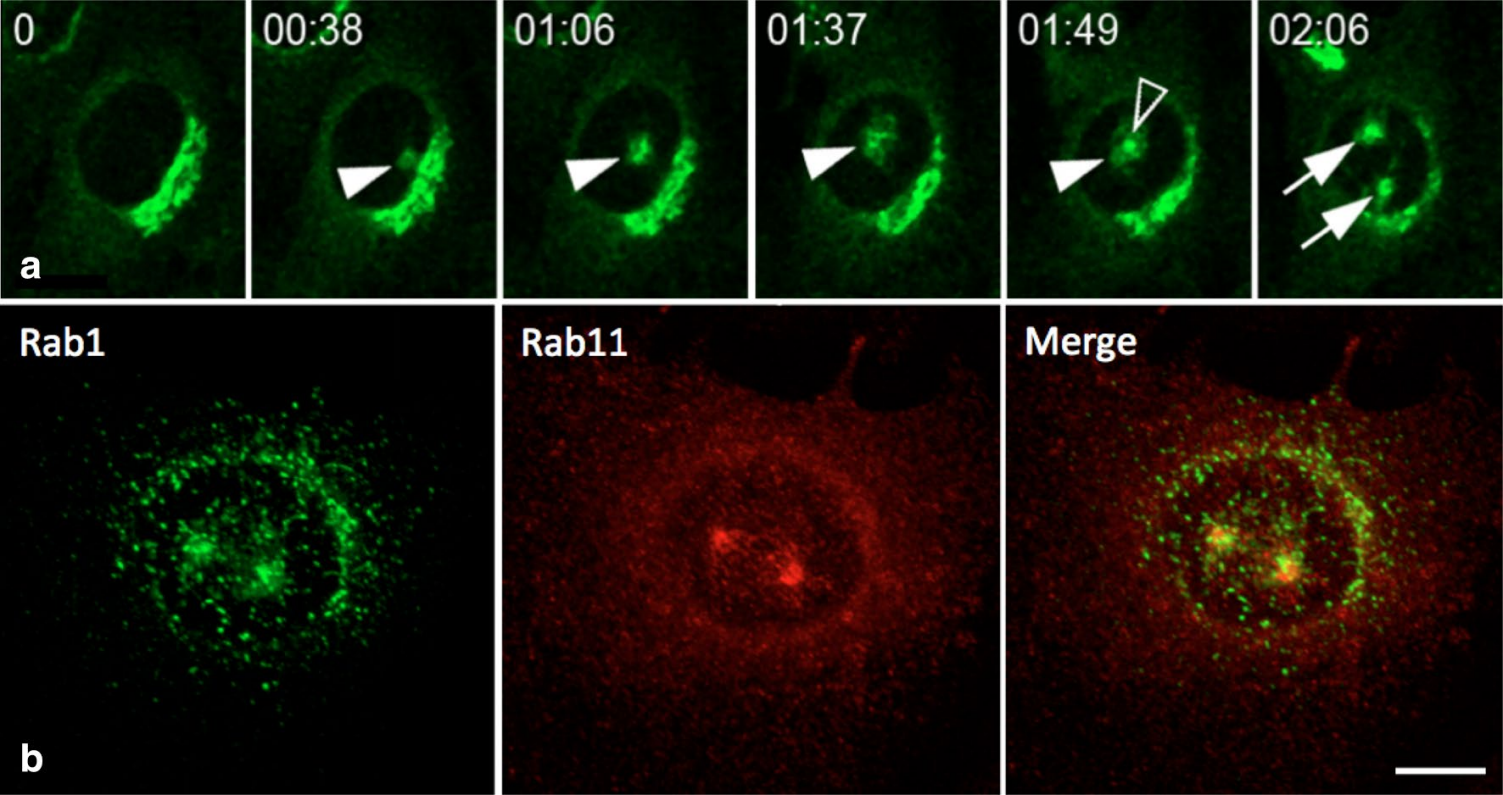
Simultaneous division of the Rab1- and Rab11-containing pericentrosomal compartments, BRC and ERC, at the onset of mitosis. **a** Selected images from a time-lapse movie (time h:min) showing an NRK cell expressing GFP-Rab1 and progressing from interphase (G_2_) to mitosis. The BRC (white arrowheads) separates from the Golgi ribbon, undergoes first expansion and then compaction (open arrowhead), and finally divides at prophase (arrows), based on the movement of the centrosomes to form the spindle poles. **b** Double staining of a GFP-Rab1-expressing NRK cell undergoing prophase with antibodies against Rab11 reveals joint division of the BRC and ERC. Reproduced from Marie et al. (2012). Bar: 10 µm

The separation of the BRC from the Golgi ribbon (Fig. 6a) allowed the identification of the entry sites of the IC elements arriving at the cell center from peripheral ERES. Namely, instead of moving directly to *cis*-Golgi, these mobile vacuoles and tubules are targeted to the pericentrosomal BRC (Fig. 5). Furthermore, imaging of IC dynamics in BFA-treated cells—following the disassembly of the Golgi stacks—revealed ongoing communication between the peripheral ERES and the BRC, which persists in these cells (Marie et al. 2009) and maintains its connection with the ERC. These results confirmed that the dynamic IC network, besides docking at ERES, is also anchored at the cell center due to its connection with the centrosome and the endocytic recycling system. Moreover, imaging of GFP-Rab1 dynamics in motile NRK cells, showing bi-directional movements of IC vacuoles and tubules between peripheral ERES and the cell’s leading edge (Sannerud et al. 2006), provided evidence that the IC is also anchored at the cell periphery. Evidently, a widespread dynamic membrane system, like the IC, could become permanent due to its anchoring at the peripheral and central end stations of the cytoskeletal MT tracks that allow bi-directional motility of its two types of constituents—relatively stationary vacuoles and highly dynamic tubules, which bud off and fuse with the vacuoles.

The idea of a stable IC received strong support from studies of mitotic cells. As cells enter mitosis, ER exit is blocked and the Golgi apparatus undergoes reversible disassembly. By contrast, the Rab1-containing IC elements persist, maintaining their compositional properties and association with the MT-based mitotic spindle. Accordingly, they are present both at the cell periphery and around the centrosomes at the spindle poles (Fig. 2). Interestingly, at mitotic prophase the BRC expands and divides in a process that is coupled to the re-positioning of the centrosomes and their movement to the spindle poles (Fig. 6). Thus, by demonstrating its autonomy from the ER and Golgi, as well as its special inheritance strategy, the IC is likely to constitute a permanent membrane system with self-organizing capability (Marie et al. 2012).

### Comparing the models

Regarding Models B and C (Fig. 4), although the dynamic features of the IC revealed by the two reporters GFP-ERGIC-53 or GFP-Rab1 were otherwise very similar, a major difference was the distinct mobilities of the large IC elements. One possible answer to this puzzle could be that GFP-Rab1 associates with the ACs that detach from the stationary IC clusters proposed in Model B. However, the notion that Rab1 is recruited to the ERES-associated IC elements in an oscillatory and BFA-resistant fashion (Sannerud et al. 2006; Marie et al. 2009), and does so to facilitate their fusion with COPII vesicles (Allan et al. 2000), makes this scenario unlikely. Another possibility is that the expression and mis-sorting of GFP-ERGIC-53 affects the maturation of the IC elements and/or their ability to gain transport competence—for example, by interfering with motor recruitment. It is also worth noting that, besides membrane-bound and soluble cargo (VSV-G and procollagen), a variety of other fluorescent reporters—including VIP36, which is closely related to p58/ERGIC-53/LMAN1—have demonstrated the mobility of the large IC elements (Blum et al. 1999; Chao et al. 1999; Dahm et al. 2001; Monetta et al. 2007).

The two models proposing the function of the IC elements as mobile transport carriers (Models A and C) are compatible with their proposed maturation, in contrast to Model B depicting a stationary IC. As discussed above, extensive membrane recycling is expected to result in the creation of compositional differences between the peripheral and central IC elements. In analogy to endosomes, the luminal conditions of the IC elements could gradually change, resulting in the establishment of a pH gradient (Palokangas et al. 1998). Acidification could also depend on the connection of the IC elements and endosomes, a possibility incorporated in Model C (see below), and affect their function in protein sorting, for example, via the recruitment of special types of COPI coats (Aniento et al. 1996). In addition, maturation via membrane recycling has been proposed to lead to the concentration of secretory proteins in the lumen of the IC elements (Martínez-Menárguez et al. 1999; Oprins et al. 2001). According to the “dynamic IC models” (Models A and C), the formation of containers for soluble cargo follows simple one-step logistics, while the “stationary IC model” (Model B) suggests a more complicated process.

Obviously, the three IC models propose very different landscapes for the transport machineries operating at the ER–Golgi interface. Regarding COPI vesicles, *Model A* emphasizes their prominent role in retrograde transport to the ER, while the two “stable IC models” (Models B and C) open up for an additional anterograde function, a view that has been revitalized by recent studies (Park et al. 2015). Alternatively, COPI coats could function in the formation of the ACs from the stationary IC clusters (Model B; Appenzeller-Herzog and Hauri 2006), or in anterograde transport from the central IC elements to *cis*-Golgi (Model C). Supporting the latter possibility, the BRC contains a separate pool of COPI (Marie et al. 2009). Model C also suggests a possible additional function of COPI vesicles in retrograde trafficking within the permanent IC network; namely, while both p58/ERGIC-53 and KDEL-R employ COPI vesicles in their trafficking, their recycling itineraries differ. Thus, during mitosis, due to the establishment of ER exit block, p58 is arrested in the ER, while KDEL-R remains as an IC component (Marie et al. 2012; Fig. 2b).

Regarding other transport factors, the two IC-associated Rab proteins, Rab1, Rab2, and their effector proteins, many of which function in membrane tethering, have been discussed elsewhere (Saraste 2016). Generally, the IC localization of the latter—for example, GM130 (Fig. 1; see also Marra et al. 2007)—opens up for their participation in homotypic fusion events within the permanent IC network, or in transport between central IC elements and the Golgi stacks (Model C).

Finally, the three models present quite different views on the relationship between the IC and the *cis*-Golgi compartment (Fig. 4). In Model B, the peripheral stationary IC clusters and the *cis*-Golgi *cisternae* are considered as distinct entities. By contrast, Models A and *C* both assume a close biogenetic relationship between the two compartments, and thus are compatible with the notion that IC residents are typically also found in the *cis*-most Golgi *cisterna*. However, these models depict the connection between IC and *cis*-Golgi in different ways: In Model A transient IC elements generate *cis*-Golgi morphology in a maturation process, while Model C raises the possibility that the *cis*-Golgi is an extension of the permanent IC network.

## Novel functions of the IC

Most of the well-characterized IC proteins are components of the transport machinery, supporting the view that the major—if not the only—task of this compartment is to carry out protein sorting and trafficking. However, over the years the IC has been assigned a variety of biosynthetic functions, such as protein folding and quality control, initiation of O-glycosylation, modification of N-linked high-mannose oligosaccharides and synthesis of glycosaminoglycans and lipids (Alvarez et al. 1999; Breuza et al. 2004; Jönsson et al. 2003; Krijnse-Locker et al. 1994; Sannerud et al. 2006; Sirkis et al. 2017; Tooze et al. 1988), indicating that its biosynthetic activities could have been underappreciated. Notably, recent studies have revealed the participation of the IC in cellular events that are not directly related to ER–Golgi trafficking. Moreover, since some of these novel tasks suggest complex interactions with the endosomal system, they are better suited for a permanent than a transient compartment.

### Golgi bypass

The intimate connection between the centrosome-linked tubular networks, BRC and ERC, is maintained not only in BFA-treated cells (Marie et al. 2009), but also during the different phases of mitosis, when the Golgi is disassembled in a controlled and reversible manner (Marie et al. 2012; Szigetvari et al. unpublished results; see Fig. 6b). Notably, various lipids and membrane proteins—including cholesterol, adhesion proteins, ion channels and GPI-anchored proteins—are delivered to the cell surface in the presence of BFA (Baldwin and Ostergaard 2002; Gee et al. 2011; Martin et al. 2001; Schotman et al. 2008; Tveit et al. 2009; Urbani and Simoni 1990), raising the possibility that they employ a direct IC-to-recycling endosome route during their Golgi-independent trafficking (Marie et al. 2008; Prydz et al. 2013). Indeed, in accordance with the suggested role of an endosomal intermediate in unconventional trafficking of the cystic fibrosis transmembrane conductance regulator (CFTR), a chloride channel (Yoo et al. 2002), evidence was obtained that this protein passes through the pericentrosomal compartments on its way to the PM (Marie et al. 2009). Conversely, the stable IC–endosome link could explain how certain molecules entering the endosomal system from the PM, such as bacterial toxins (Sandvig et al. 1992), can gain access to the early compartments of the secretory pathway.

Recently, the idea of a Golgi bypass based on direct communication between the IC and the endocytic recycling system received strong support from an unexpected source, namely, the study of protein trafficking in neuronal dendrites. Remarkably, hundreds of transmembrane proteins of the synaptic PM—including neurotransmitter receptors, ion channels and adhesion proteins—that are locally synthesized at the neuronal periphery can reach the cell surface containing high-mannose oligosaccharides, indicating that they are not modified by Golgi enzymes. Moreover, cell surface expression of these proteins is not affected by BFA (Hanus et al. 2016). Furthermore, it turns out that most dendrites lack the so-called Golgi outposts, but do contain IC elements and recycling endosomes (Hanus et al. 2014; Krijnse-Locker et al. 1995; Sannerud et al. 2006). Thus, proteins leaving the dendritic ER are delivered to the surface of synaptic spines via a bypass route that connects these two compartments (Bowen et al. 2017). An important implication of these results is that similar “IC-recycling endosome units”—that may be more readily identifiable at the neuronal periphery—also exist in the densely packed perinuclear endomembrane system in the cell bodies. Evidently, Golgi bypass is not an exception to the rule—limited to a set of unconventional molecules—but an important general mechanism for polarized cell surface transfer of proteins and lipids (Prydz et al. 2008; Saraste et al. 2009)—at least in certain cell types and physiological situations.

The mechanism(s) of molecular exchange between the biosynthetic (IC) and endosomal networks remains an open question. Since Rab1 and Rab11 define these compartments (Saraste and Goud 2007), they are good candidates to regulate these transfers, as well. Indeed, many of the effectors of the IC-associated GTPases Rab1 and Rab2—such as GMAP210, p115 and GM130 (Fig. 1**)**—act in membrane tethering and also localize to the BRC (Roboti et al. 2015; Saraste 2016). Classical mechanisms involving COPI or clathrin coats are probably excluded, since the transfer is not affected by BFA. However, EM analysis revealed that the pericentrosomal tubules contain BFA-resistant coats (Tooze and Hollinshead 1992). Interestingly, many of the proteins displaying BFA-resistant trafficking have been implicated as components of lipid rafts (Marie et al. 2008), suggesting that IC–endosome communication may be based on lipid-mediated sorting and trafficking (Zurzolo and Simons 2015). Also, the two types of tubules could establish membrane contact sites for the exchange lipids.

### Autophagosome biogenesis

Interestingly, evidence for Rab1- and Rab11-dependent communication between the IC and recycling endosomes was provided by studies of autophagy. The formation of autophagosomes that sequester cytoplasmic material for degradation in lysosomes involves multiple organelles and depends on membrane traffic. Interestingly, recent studies have uncovered the important roles of the IC and recycling endosomes in this multistep process. Cell-free reconstitution revealed that the IC is an important membrane source for autophagosome formation (Ge et al. 2013). Surprisingly, both COPII and COPI vesicles have been suggested to deliver membrane from the IC/*cis*-Golgi to the forming autophagosome during the different stages of its assembly (Ge et al. 2017; Karanasios et al. 2016). In addition, Rab1 and Ypt1 have been identified as important regulators of autophagy in mammalian and yeast cells, respectively (Huang et al. 2011; Lipatova et al. 2012; Lynch-Day et al. 2010; Mochizuki et al. 2013; Winslow et al. 2010; Zoppino et al. 2010). Interestingly, their functions are related to another membrane source for autophagosome biogenesis, that is, *trans*-Golgi/endosomal vesicles marked by the transmembrane protein Atg9. In yeast, the recruitment of Atg9 vesicles to the site of autophagosome formation depends on the activation of Ypt1 by TRAPPIII (Kakuta et al. 2012). In mammalian cells, the delivery of Atg9 from recycling endosomes to the IC/*cis*-Golgi was shown to depend on a molecular link between Rab11 and Rab1, which leads TRAPPIII-mediated activation of the latter (Lamb et al. 2015). Thus, a similar autophagy-related activation mechanism of Rab1/Ypt1, involving coupling between the IC and *trans*-Golgi/endosomal compartments, seems to operate in both yeast and mammals.

### Protein maturation

By retrieving incompletely folded or assembled proteins in COPI-vesicles the IC/*cis*-Golgi system collaborates with the ER in a quality control process ensuring that only mature proteins reach the Golgi apparatus (Anelli and Sitia 2008; Barlowe and Helenius 2016, Hsu et al. 1991; Roth and Zuber 2017). Accordingly, the IC is the site of localization of glucosidase II and UDP-glucose: glycoprotein glucosyltransferase, key components of the quality control machinery (Lucocq et al. 1986; Zuber et al. 2001). In addition, the KDEL-containing molecular chaperones present in the IC—such as BiP, PDI and calreticulin (Breuza et al. 2004; Zuber et al. 2001)—could cycle bound to their unfolded client proteins, enabling post-ER quality control (Hammond and Helenius 1994). For example, ERp44 (a PDI family member) and BiP interact in the IC/*cis*-Golgi in a pH-dependent manner with the KDEL-receptor, resulting in ER retrieval of their clients, unassembled IgM or T-cell antigen receptor subunits, respectively (Vavassori et al. 2013; Yamamoto et al. 2001). Moreover, a recent study demonstrated the COPI-dependent cycling of neurodegeneration-related mutant membrane proteins in the early secretory pathway (Sirkis et al. 2017). Additional evidence for the function of the IC/*cis*-Golgi as a post-ER quality control checkpoint is provided by the observed accumulation misfolded proteins in the IC elements (Hermosilla et al. 2004; Raposo et al. 1995; Zuber et al. 2004).

### Signaling

A number of protein kinases have been implicated in the regulation of COPI-dependent retrograde trafficking at the IC/*cis*-Golgi level (Hamlin et al. 2014; Tisdale and Artalejo 2006). Notably, the KDEL-receptor—a seven-spanning transmembrane protein reminiscent of cell surface G protein-coupled receptors—is a key component of a homeostatic control system that regulates antero- and retrograde trafficking at the ER–Golgi interface and across the Golgi stacks (Cancino et al. 2014). The receptor senses incoming transport fluxes due to binding of KDEL-proteins, leading to interaction with two different Golgi-associated heterotrimeric G-proteins. The latter activate Src-kinases, which regulate the transport machineries via phosphorylation (Giannotta et al. 2012; Pulvirenti et al. 2008). Recently, the interaction of the KDEL-receptor with still another G-protein was shown to activate a conserved signaling path that involves Rab1 and controls the formation of cellular protrusions, such as neurites (Solis et al. 2017). This finding in relevant in terms of the previously demonstrated, Rab1-mediated Golgi bypass route from the IC to the neurite-like extensions and growth cones of PC12 cells (Sannerud et al. 2006).

## Implications of the stable IC–endosome connection for Golgi biogenesis

The separation and pericentrosomal expansion of the BRC and ERC in motile or dividing normal rat kidney (NRK) cells (Marie et al. 2009, 2012, Fig. 6) coincide with the fragmentation of the Golgi ribbon (Acharya et al. 1998; Bisel et al. 2008). Based on this observation we proposed that these compartments are derived from the tubulovesicular networks that connect the different Golgi stacks (Marie et al. 2012). This is in accordance with EM tomographic data on the organization of the Golgi ribbon in NRK cells, showing that these “non-compact regions” contain pleiomorphic structures resembling IC elements or endosomes (Ladinsky et al. 1999). In addition, the existence of a Golgi bypass pathway in neuronal dendrites (Bowen et al. 2017) revealed that the functional connection between the IC elements and recycling endosomes does not depend on their close association with the centrosome.

**Fig. 7:**
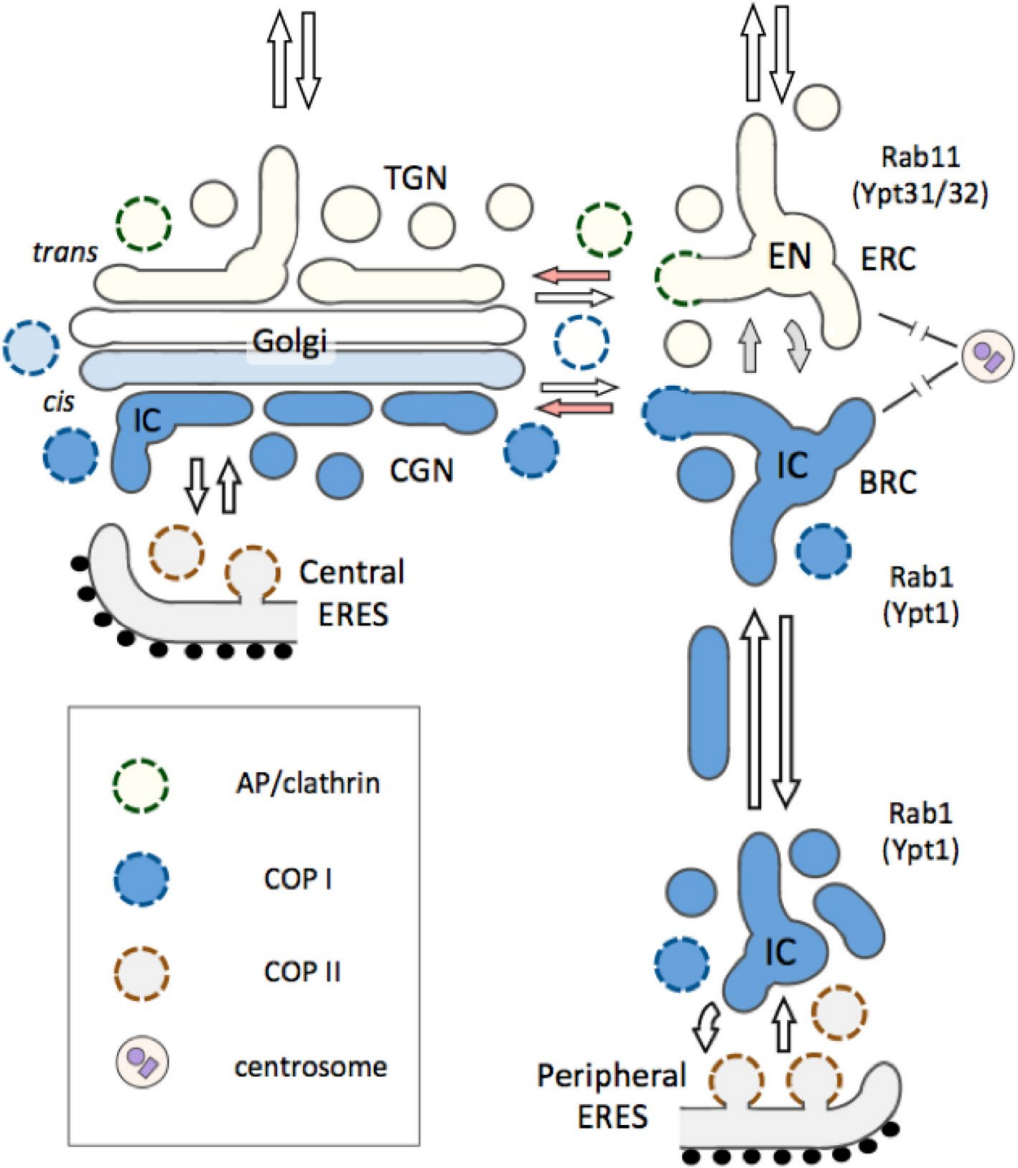
Spatial and functional relationship of the biosynthetic (IC) and endosomal (EN) networks with the Golgi stacks. According to this model the “mirror compartments” BRC and ERC (Saraste and Goud 2007) normally exist within the Golgi ribbon at some distance from the centrosome. These tubular compartments develop from IC and endosomal structures that occupy the non-compact regions connecting the different Golgi stacks, but expand and accumulate around the centrosome, when the latter separates from the Golgi ribbon—for example, due to cell motility or division. In addition, the IC elements and recycling endosomes at the linker regions communicate bi-directionally with the Golgi stacks, perhaps preferentially with the tubular networks at the *cis*- and *trans*-sides of the organelle (CGN and TGN). The red arrows indicate Arf1-regulated, COPI- or clathrin-dependent transport steps, which are blocked by BFA. The same transport steps could be inhibited at the onset of mitosis due to phosphorylation of key regulatory components, leading to Golgi fragmentation. The Rab GTPases regulating transport at the level of the BRC and ERC (Rab1 and Rab11), and the early and late Golgi compartments of *S. cerevisiae* (Ypt1 and Ypt31/32) have been indicated, suggesting a close relationship of the tubular networks operating in the mammalian and yeast secretory pathways (Jackson 2009; Marie et al. 2008)

The BFA-resistance and mitotic preservation of the IC elements and recycling endosomes suggest that they play key roles in Golgi biogenesis. The schematic model in Fig. 7 proposes that these stable compartments, occupying the linker regions of the Golgi ribbon, generate cisternal organization at the two sides of the Golgi stacks in a process that depends on the BFA-sensitive COPI and clathrin coats. Regarding the *trans*-side, the model is based on the existence of a AP-1/clathrin-dependent transport route from recycling endosomes to the *trans*-Golgi/TGN (Hinners and Tooze 2003; Lu and Hong 2014; Mallard et al. 1998; Matsudaira et al. 2015). Regarding *cis*-Golgi events, the model takes into account the function of COPI-coated tubules in anterograde transport at the level of the Golgi stacks (Park et al. 2015). Notably, the integrity of the Golgi ribbon depends on the formation of these tubules (Yang et al. 2011). As indicated in Fig. 7, disassembly of these coats by BFA blocks the two pathways (red arrows), resulting in rapid breakdown of the Golgi stacks. By contrast, the BFA-resistant “linker compartments”—defined by Rab1 and Rab11—persist and pile up around the centrosome. The BFA-induced redistribution of *cis*- and *medial*-Golgi enzymes, such as mannosidase II, to the ER occurs via the pericentrosomal IC tubules (Marie et al. 2009), supporting this model. On the other hand, TGN markers—such as furin and TGN38—that cycle between *trans*-Golgi/TGN and the endosomal system accumulate around the centrosome in BFA-treated cells, suggesting that their normal retrieval from recycling endosomes to the TGN is blocked (Molloy et al. 1994; Reaves and Banting 1992). Thus, coat-independent pathways remain operational in BFA-treated cells, resulting in the redistribution of Golgi components. The COPI-independent tubular pathway that is regulated by Rab6 may participate in these events (Heffernan and Simpson 2014; Sengupta et al. 2015).

The model in Fig. 7 is also supported by the data on the localization of the BFA-sensitive GEFs, GBF1 and BIG1/BIG2, that activate Arf1 at the two sides of the Golgi ribbon, thereby regulating the recruitment of COPI and clathrin coats. Namely, GBF1 is predominantly localized to the IC/*cis*-Golgi, while BIG1/BIG2 are present in both recycling endosomes and *trans*-Golgi/TGN (Ishizaki et al. 2008; Shen et al. 2006; Togawa et al. 1999; Zhao et al. 2006). Indeed, previous studies demonstrated that the knock-down of GBF1 and BIGs results in disassembly of the Golgi stacks and the *trans*-Golgi/TGN, respectively (Manolea et al. 2008).

The present model could also apply to the events that take place at the onset of mitosis (Ayala and Colanzi 2017). The phosphorylation of key machinery proteins at the G_2_/M transition—such as GRASP55, GRASP65 and possibly also BARS—could affect the same step(s) as BFA (Fig. 7, red arrows), but in this case resulting initially in the fragmentation of the Golgi ribbon. Notably, mitotic progression could depend on proper detachment of the linker compartments from the Golgi ribbon and their accumulation around the centrosome (Fig. 6; Marie et al. 2012).

The relationships of the compartments at the *cis*- and *trans*-sides of the Golgi—the IC and *cis*-Golgi/CGN and recycling endosomes and the *trans*-Golgi/TGN, respectively—have been difficult to define, due to their spatial overlap and compositional and functional similarities. The biogenetic relationship between these compartments, proposed in Fig. 7, can explain why machinery proteins operating at the level of pre-Golgi IC or post-Golgi recycling endosomes are also invariably found at the *cis*- and *trans*-sides of the Golgi stacks. The placement of the IC and recycling endosomes as linker compartments in the Golgi ribbon could also clarify the spatial relationship between pathways that involve classical Golgi passage or bypass the cisternal stacks. In addition, the “Golgi bypass route” depicted in Fig. 7 could be utilized to generate the polarized distribution of Golgi components (Marie et al. 2008). It could also give rise to aberrant cell surface delivery of proteins under conditions when their normal routes are compromised. Furthermore, the model in Fig. 7 opens up the possibility that COPI vesicles at the non-compact regions (Ladinsky et al. 1999; Rabouille and Klumperman 2005; Yang et al. 2008) mediate the cycling of Golgi enzymes between the linker compartments and the Golgi stacks (Alvarez et al. 1999; Jarvela and Linstedt, 2011). Encountering the mildly acidic luminal milieu of the linker compartments (Palokangas et al. 1998; Yamashiro et al. 1984) could regulate the assembly of the glycosyltransferase complexes (Hassinen and Kellokumpu 2014).

Finally, the present model on the role of the interconnected biosynthetic (IC) and endocytic tubular networks in the biogenesis of the Golgi stacks can explain the relationship between the yeast and mammalian secretory systems. Accordingly, the yeast pathway consisting of tubular networks, some with combined secretory and endocytic activities (Day et al. 2018), is likely to represent a primordial endomembrane system that is employed by mammalian cells as the basis for the build-up of the Golgi ribbon (Fig. 7; Jackson 2009; Marie et al. 2008). An obvious difference is that the tubular networks in the mammalian cells have been linked to the radiating MT system that is anchored to the centrosome.

## Summary and perspectives

Based on recent imaging studies, the main message of this review is that the IC—instead of corresponding to a collection of transient transport carriers operating between the ER and the Golgi stacks—represents a permanent membrane system that extends throughout the cytoplasm. The stable nature of the IC is supported by recent findings showing that this compartment has multiple functions that are not all necessarily related to ER–Golgi communication. Moreover, the behavior of the IC during mitosis shows its stability and autonomy from the ER and Golgi. Notably, however, the joint division of the BRC and ERC at the onset of mitosis (Fig. 6) demonstrates the close partnership of the biosynthetic (IC) and endocytic tubular networks, which also appear to communicate during interphase. Indeed, we can see the BRC–ERC link again at the cell center, when these compartments pile up around the centrosome during Golgi repositioning in motile cells (Marie et al. 2009; Dale et al. in preparation). Also, recent studies indicate that they join forces at more peripheral sites, possibly including the Golgi ribbon, as suggested in Fig. 7. An intimate spatial and functional connection between these networks could expain mutant cells, where both secretion and endocytosis are affected (Gao et al. 1994; Kao and Draper 1992), as well as the appearance of IC/*cis*-Golgi proteins in “wrong” places at the outskirts of the cell (Kaczmarek et al. 2017; Sannerud et al. 2006).

Morphological studies employing an ever-increasing repertoire of advanced light and electron microscopic techniques, including super-resolution microscopy, can be expected to also play a key role in future studies aimed at the elucidation of the functional organization of the IC and its relation with the Golgi stacks and the endosomal system. As demonstrated by recent studies on neurons (Bourke et al. 2018; Bowen et al. 2017; Hanus and Ehlers 2016; Mikhaylova et al. 2016), examination of specialized cell types, such as epithelial and muscle cells, can also help to reveal additional secrets of this fascinating membrane system.
